# Investigation of Alkali-Activated Slag-Based Composite Incorporating Dehydrated Cement Powder and Red Mud

**DOI:** 10.3390/ma16041551

**Published:** 2023-02-13

**Authors:** Aref A. Abadel, Hussam Alghamdi, Yousef R. Alharbi, Mohammed Alamri, Mohammad Khawaji, Mohammed A. M. Abdulaziz, Moncef L. Nehdi

**Affiliations:** 1 Department of Civil Engineering, College of Engineering, King Saud University, Riyadh 11421, Saudi Arabia; 2 Department of Civil Engineering, McMaster University, Hamilton, ON L8S 4M6, Canada

**Keywords:** slag, geopolymer, compressive strength, microstructure, curing, dehydrated cement powder

## Abstract

Recycled construction cementitious materials (RCCM) and red mud (RM) could be considered a type of discarded material with potential cementitious properties. Generally, landfilling and stacking are utilized to dispose of this type of solid waste, which can be detrimental to the environment and sustainability of the construction sector. Accordingly, a productive process for making eco-efficient alkali-activated slag-based samples with the inclusion of RCCM and red mud is studied in this paper. Dehydrated cement powder (DCP) is attained through the high-temperature treatment of RCCM, and red mud can be obtained from the alumina industry. Subsequently, DCP and RM are utilized as a partial substitute for granulated blast furnace slag (GBFS) in alkali-activated mixtures. Two different batches were designed; the first batch had only DCP at a dosage of 15%, 30%, 45%, and 60% as a partial substitute for GBFS, and the second batch had both DCP and RM at 15%, 30%, 45%, and 60% as a partial substitute for GBFS. Different strength and durability characteristics were assessed. The findings show that when both dehydrated cement powder and red mud are utilized in high quantities, the strength and durability of the specimens were enhanced, with compressive strength improving by 42.2% at 28 days. Such improvement was obtained when 7.5% each of DCP and RM were added. The results revealed that DCP and RM have a negative effect on workability, whilst they had a positive impact on the drying shrinkage as well as the mechanical strength. X-ray diffraction and micro-structural analysis showed that when the amount of DCP and RM is increased, a smaller number of reactive products forms, and the microstructure was denser than in the case of the samples made with DCP alone. It was also confirmed that when DCP and RM are used at optimized dosages, they can be a potential sustainable binder substitute; thus, valorizing wastes and inhibiting their negative environmental footprint.

## 1. Introduction

Portland cement is the most highly utilized construction material around the globe. The emission of CO_2_ from the manufacturing of Portland cement is nearly 5 to 7% of the worldwide release of carbon dioxide [1,2,3,4], which has detrimental impacts on the world’s response to environmental protection [5]. Hence, environmental activists and concerned officials in different nations have been inspiring the cement sector to decrease its release of carbon dioxide [6,7,8]. There are two methods of reducing the utilization of cement, the 1st is the usage of blended cement, and the 2nd is the usage of alkali-activated granulated blast furnace slag (GBFS) [9], rice husk ash (RHA) [10], fly ash (FA) [11], palm ash [4,12,13], or any other material with rich aluminosilicates [14]. Using alkali-activated materials [11] uses less energy and decreases carbon dioxide’s outflow. Additionally, alkali-activated materials improve concrete durability and assist in solving the issue of landfilling [15,16]. Various eco-efficient alkali-activated pastes (AAP) have been investigated and developed, leading to significantly reduced CO_2_ [17]. Binders use industrial by-products such as silica fume, fly ash, and GBFS to create GPs when making concrete or pastes [18]. Industrial by-product materials have also been used in many concrete applications at a micro- and nano-scale [19,20,21]. These products can lead to sustainable material with a reduced outflow of carbon dioxide compared to Portland cement. Researchers and commercial sectors currently focus on producing Portland cement substitutes [22]. The properties and abilities of such a substitute to decrease the outflow of carbon dioxide and have high performance against fire and chemical attacks while at the same time signifying good strength and functionality parallel to Portland cement are amid the domains that have drawn the consideration of researchers. The utilization of multiple alkali chemical activators (ACA) instigates a chemical reaction from different materials that have high aluminosilicates, and this research domain with the microstructural analysis of such materials is the target research area of different studies [23,24]. As of now, the number of mixes for eco-efficient paste, mortar or concrete has not been developed much. During the past years, a huge amount of research has been conducted concerning the development of sustainable paste, concrete, and mortar [25,26].

The use of fly ash as a source material for forming an alkali-activated binder stands out from other source materials. Many amorphous SiO_2_ and Al_2_O_3_ exist within fly ash, which develops from electricity production in thermal plants when coal makes fly ash a waste product [27]. Hence, the suitability of fly ash as the primary material for making alkali-activated binders is confirmed by its chemical arrangement. Various researchers have studied the characteristics and aspects of fly ash-based alkali-activated binders and their utilization as potential binding materials due to their incredible durability [28]. Wang et al. [29,30] investigated the effect of fly ash dosage on the permeability and shrinkage in concrete slabs. The results show that the addition of 30% fly ash dosage was optimal for refining the pore structures and reducing shrinkage for face slab concretes, without compromising much of the mechanical property. The engineering characteristics of fly ash make it well-matched for various construction activities. Though fly ash-based alkali-activated binders have some problems because of their lesser compression strength, a heat of 45 to 90 °C is required for the curing samples and slower setting time [31]. The general characteristics of such alkali-activated binders can be optimized with the inclusion of another industrial discarded material, for instance, granulated blast furnace slag, to overcome these problems. Shapeless hydrated alkali aluminosilicate and C-S-H are the primary products of the reaction of alkali-activated binders formed from fly ash and granulated blast furnace slag [32]. Dry shrinkage, low slump value, and issues with fast setting time influence alkaline-activated granulated blast furnace slag, limiting its utilization regardless of higher compression strength [16]. These low strength problems, setting time, and slump value can be overcome by including granulated blast furnace slag in alkali-activated binders. The durability properties of alkaline-activated paste or mortar in acid surroundings could be reduced because of the granulated blast furnace slag [11]. Furthermore, a reduction in a mortar or paste’s resistance against aggressive surroundings is triggered by the vast proportion of calcium oxide in granulated blast furnace slag [33].

Red mud waste is created from the aluminum sector due to the Payer method for mining alumina from the ore of bauxite, which comprises treating the ore of bauxite with a chemical called NaOH [34]. The development of red mud is projected to be between 1.4 tons to 2.6 tons per ton of alumina, depending on the origin and efficiency of the mining method [35]. Most of these contaminants are landfilled and discharged into the sea when treated [36,37]. Because of its high alkalinity and capability of cation exchange, storing red mud (RM) covers a large area of space. It is considered a significant threat to the ground, surroundings, sea, and groundwater [38]. Dealing with harmful elements from red mud can harm the ocean and groundwater. Hence, discarding red mud safely is an essential concern for the alumina industry, based on its huge consumption of raw materials and significant impact on the atmosphere. Alkaline-activated binders are a type of binding material that could be formed from industrial discarded materials with high volumes of alumina-silicates. They are considered a replacement material for traditional Portland cement [39]. The utilization of RM in the production of alkali-activated cement and cement clinker is reported by Zhang and Liu [40] and Gomes et al. [41]. These alumina-silicate elements derive from the industries’ waste materials, and geological minerals comprise alumina-silicates, such as red mud. Red mud could be utilized with other materials to make a composite binder [42].

It is a recognized fact that discarding construction and demolition waste (C&D) is considered a significant global issue [31,43]. For example, in China, the growth of urbanization instigates a considerable proportion of construction waste annually [44]. Much of this construction waste will be dumped in landfills, further prompting environmental and climate problems. Currently, many researchers focus on using recycled aggregates in concrete generated from C&D, but utilizing only recycled aggregates is insufficient [45]. Hence, recycled cementitious construction materials (RCCM) can decrease the total proportion of waste developed from C&D and also assist in the improvement of the environment [46]. One of the significant parts of RCCM is stiff cement paste, which can reinstate the capacity of hydration when exposed to a higher heat of 500 to 950 °C for a few hours, and some products of hydration in higher heat surroundings will slowly dehydrate, for example, gel of calcium silicate hydrate and calcium hydroxide. When the rehydration of dehydrated cement powder (DCP) happens, these dehydrated products of hydration will improve into novel products of hydration. The newly created products of hydration are considerably identical in behavior to the primary products of hydration. In comparison to the cement which is generally utilized, DCP can be considered a novel type [47] of eco-friendly binding material, which is highly reactive and also has minimal influence on the environment.

## 2. Experimental Program

### 2.1. Materials

#### 2.1.1. Granulated Blast Furnace Slag (GBFS)

As GBFS is an industrial by-product, this was obtained from a local place in powder form. Its chemical composition is presented in Table 1.

#### 2.1.2. Dehydrated Cement Powder (DCP)

Generally, DCP can be attained from different places, for instance, concrete from an uninhibited building, construction of a tunnel, and waste materials in laboratories. The chemical composition of DCP is presented in Table 1, as determined by X-ray fluorescence (XRF) analysis. The purpose behind DCP being selected from the testing lab is as follows; (i) Accessibility: The test labs generate a high proportion of binder-based waste every year. (ii) Economy: The discarded cementitious material obtained in this study has already been destroyed during the testing, which means that it will require less effort to develop the waste. It also helps in the cost-saving of processing the waste in the lab, and access to these materials can be attained without any extra shipping cost.

#### 2.1.3. Red Mud Powder (RM)

Red mud (RM) originated from the aluminum metallurgical plant in Saudi Arabia and was dried to constant mass at a temperature of 105 °C and then ground in a ball mill. RM powder samples were calcined in a furnace under static air at a heating rate of 10 °C/min until temperatures of 750 °C were attained. The samples were kept at these temperatures for two hours. The calcined samples were then removed from the furnace and allowed to cool naturally to room temperature in the air. The chemical properties, as determined by X-ray fluorescence (XRF) analysis, are tabulated in Table 1.

#### 2.1.4. Alkali Solutions

Two different types of alkaline chemicals were used in this study. The first was sodium hydroxide with a purity level of 99%, and the second solution was sodium silicate.

### 2.2. Mix Proportion and Preparation of Specimens

By making five different types of mixes, the influence of the utilization of DCP as a fractional replacement for GBFS on the characteristics of alkali-activated paste was observed. Complete details of mix proportioning can be observed in Table 2. Two different batches of modified samples were developed.

In the first batch, DCP was added to the compounds in the amounts of 0%, 15%, 30%, 45, and 60% as a partial replacement for GBFS. In the second batch, DCP and RM were added by a combined 15%, 30%, 45%, and 60% as the substitute material for the GBFS. The terminology of the mix design was arranged in such a way that the “C” and “R” after “M” denotes the amount of DCP and red mud as a substitute for GBFS in the specific mix. The water-to-binder ratio was kept at 0.395. 12, 12. Samples for mechanical (compressive, flexural) strength were tested at 7, 14, and 28 days of curing. As per ASTM C 305 specifications, a mechanical stirrer was utilized to make the specimens. The pastes were emptied into a cube mold of 50 mm × 50 mm × 50 mm, and the fresh specimens were wrapped with a coating. The specimens were then positioned in curing with a temperature of 22 °C. After two days of sample preparation, the specimens were removed from the mold and sealed with a coating layer for wrapped curing at 22 °C.

### 2.3. Test Methods

**Figure 1:**
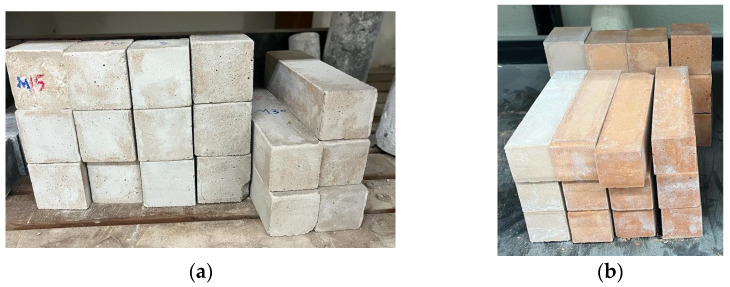
Representative samples used for testing. (**a**) DCP; (**b**) DCP + RM.

For the compressive strength test, ASTM C 109 [48] was followed. For this test, concrete cubes of 50 mm × 50 mm × 50 mm were prepared per ASTM C 579 [49] and tested at 7, 14, and 28 days of curing. For the flexural strength test, ASTM C 78 [50] was followed. Concrete beams were prepared and tested by the three-point bending method at 7, 14, and 28 days of curing. Four samples for mechanical strength were tested. Figure 1 shows representative samples used for compressive and flexural testing. As per the standard of ASTM C 1437 [51], the freshly mixed paste should be tried for a fluidity test. Cone was positioned at the center of the plate, and the paste was gradually discharged into it. Cone is then vibrated twenty times, and the mold’s top surface is smoothly polished with a scraper. The diameter of two vertical intersections was then assessed. As per the standard of ASTM C 267 [52], a ten percent sulphuric acid was used for the acid attack test on samples. After curing for 56 days, the specimens were placed in a solution of sulphuric acid and then tested for residual compressive and flexural strength at 56 days. To evaluate the dry shrinkage of samples, prisms were prepared with a dimension of 40 mm × 40 mm × 160 mm as per ASTM C 157 [53]. To determine the porosity of the samples, ASTM C 830 [54] was followed, and the samples were tested at 7, 28, and 91 days. To measure the efflorescence of the samples, the samples were positioned in water of 20 mm height for one week. Visual observations were taken. The height of the water was kept uniform during the test. The porosity of samples was assessed by following ASTM C 642 [55]. Three samples from every mix were completely dipped in a water tank with a water temperature of 22 °C for one day. Lastly, X-ray diffraction and scanning electron microscope tests were performed to understand the morphology and behavior of sample at microscopic level.

## 3. Results and Discussion

### 3.1. Flowability

**Figure 2:**
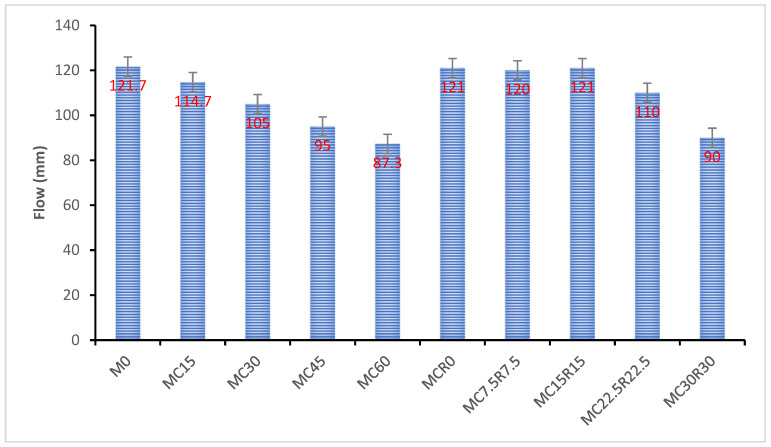
Effect of DCP content on the flowability of the mixture.

**Figure 3:**
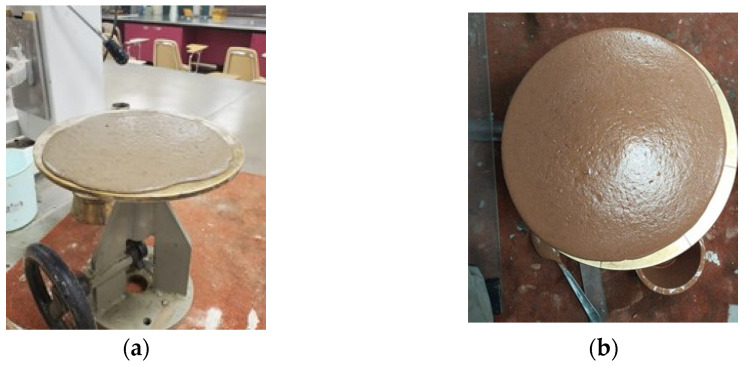
Test setup of flowability of GBFS with: (**a**) DCP; (**b**) DCP + RM.

Different factors influence the flow properties of alkali-activated paste, for instance, water-to-binder ratio, type of alkaline activator utilized, physical characteristics of materials, etc. This research measures only the effect of DCP as a substitution for GBFS on the flow. The flowability of all newly mixed samples is presented in Figure 2, while the test setup is displayed in Figure 3a,b. From Figure 2, it was noticed that as the proportion of DCP increased in samples, the flowability of newly mixed samples began to reduce. Compared to the reference sample, the flow values of samples with DCP were decreased by 5.7%,13.7%, 21.9%, and 28.2% for M15, M30, M45, and M60 mixes. The reduced flow value of alkali-activated pastes could be because of the raw ingredients’ chemical and physical characteristics [56]. Compared to GBFS, DCP offers more reactive materials, which can cause quick agglomeration and lead to samples with low values of flowability [57]. It can be noted from Figure 2 that when both DCP and RM were added to the sample, the flowability was initially enhanced significantly (21.48%) than the samples having only DCP. However, the flowability was reduced when DCP and RM were added at 30%. This could be related to the porous behavior of RM, which ingests more water and lowers the flowability. The reduction in flowability is also attributed to iron oxide’s solubility, which is more soluble in the acidic medium than in the primary medium.

### 3.2. Compressive Strength

**Figure 4:**
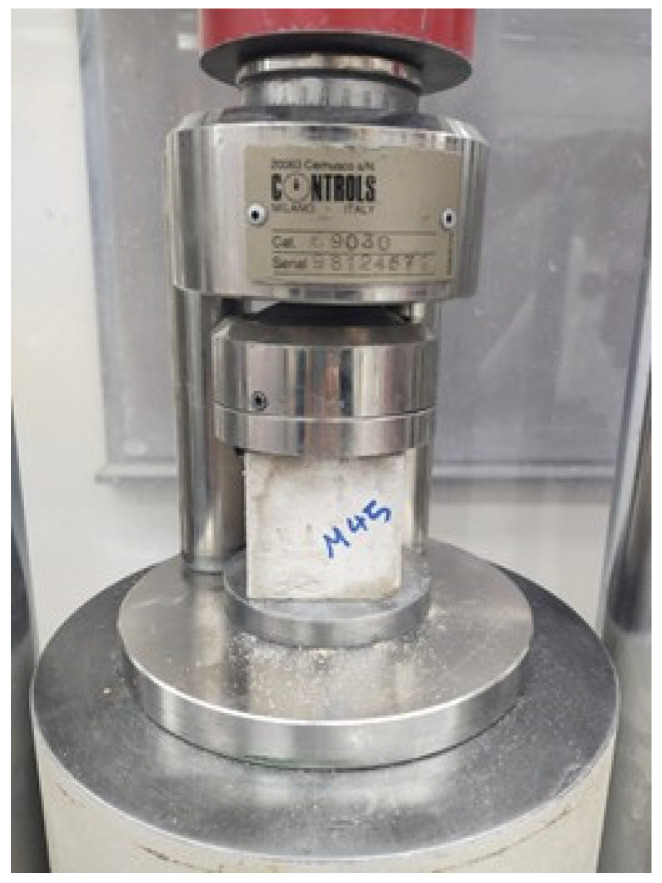
Compression testing machine.

**Figure 5:**
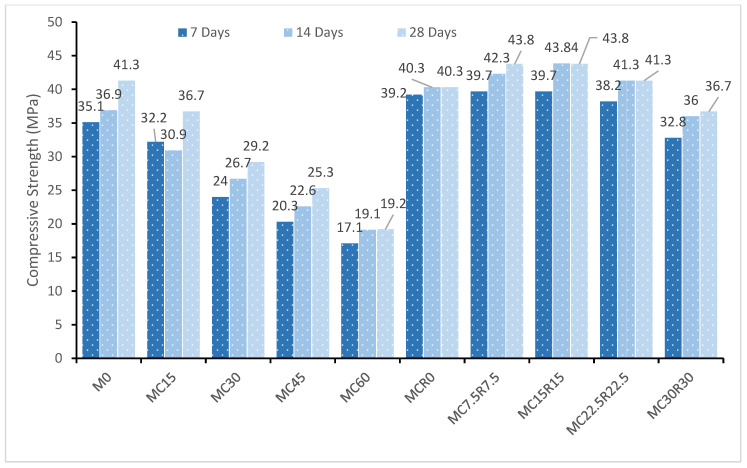
Compressive strength (MPa) of alkali-activated samples at 7, 14, and 28 days.

A compression testing machine (displayed in Figure 4) was utilized to determine the alkali-activated sample’s compressive strength. Several factors influence the compression strength of alkali-activated materials, for instance, the temperature for curing, the concentration of the alkaline solution, and the molar ratio. This paragraph assesses and debates the influence of including DCP on the compression strength of alkaline-activated pastes. The compression strength of the sample when the rate of substitution of GBFS with DCP was 15%, 30%, 45%, and 60% was measured and contrasted with a reference sample. Figure 5 depicts the outcome of the compressive strength test of alkali-activated samples. The compressive strength of samples with DCP at an early stage can be noted as lesser than the reference specimen. At 28 days, the mix “M0” displayed the highest compressive strength with 41.3 MPa, and, as the proportion of DCP was raised in samples, the compressive strength began to decrease with it. This reduction in compression strength could be ascribed to the low amount of silica [58], which changes the ratio of calcium to silica and detrimentally influences the compression strength. Additionally, it impacts the formation of calcium-aluminate-silicate-hydrate gel, which may slow the rate of early chemical reactions [59]. However, as noted in Figure 5, the samples’ later strength increased compared to its seven days strength for every mix by 15.01%, 12.2%, 17.80%, 19.76%, and 10.93% for every combination of M0, M15, M30, M45, and M60. This rise in compression strength can be credited to reactive silica in DCP, which contributed to the chemical reaction. When both DCP and RM were added as a replacement material for GBFS in the samples, the compressive strength improved considerably. At 28 days, the highest compressive strength was obtained than other curing days. At 28 days, the compressive strength was enhanced by 42.2% when both 7.5% DCP and 7.5% RM replaced the slag mixture. The major motive behind the improvement in strength is because of the existence of a high volume of hatrurite in the binder’s matrix, as also revealed by past research [60]. Reactive silica improves the process of geo-polymerization and presents additional silicon into the polymer chain, increasing the later age strength of geopolymer mixes [33]. Additionally, the sodium atoms in RM have a high impact on the structure formation and morphology of the gel of CSH over the initial stage of hydration [61].

### 3.3. Flexure Strength

**Figure 6:**
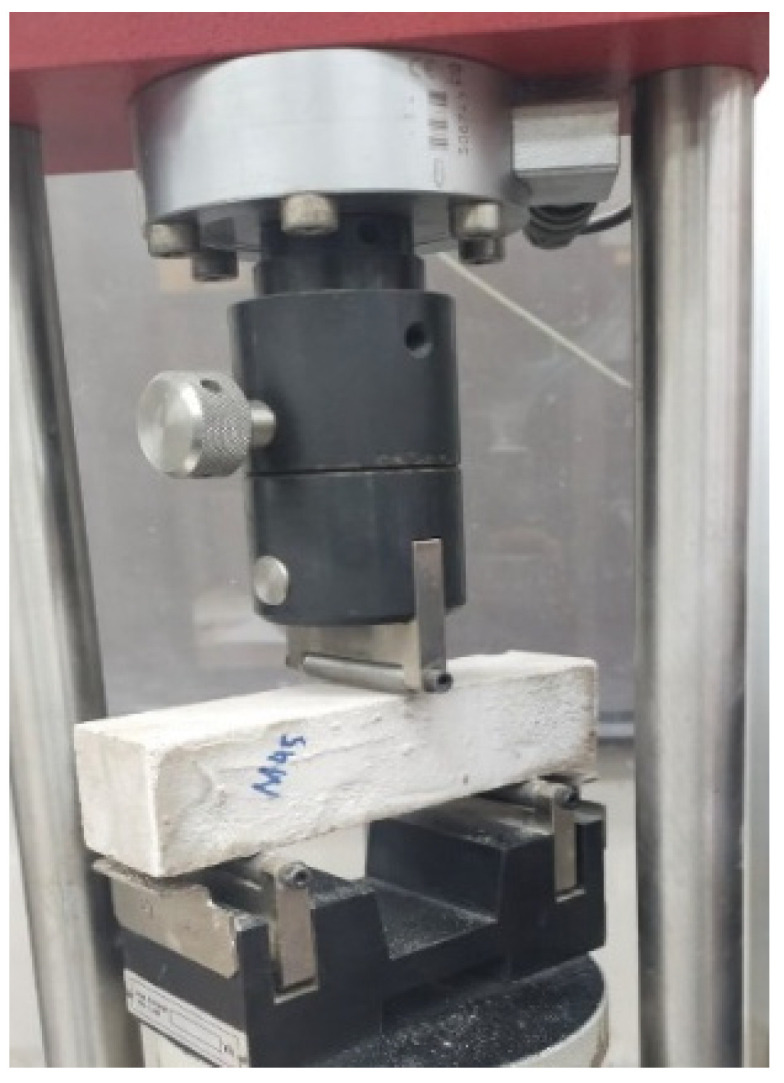
Test setup for flexural strength.

**Figure 7:**
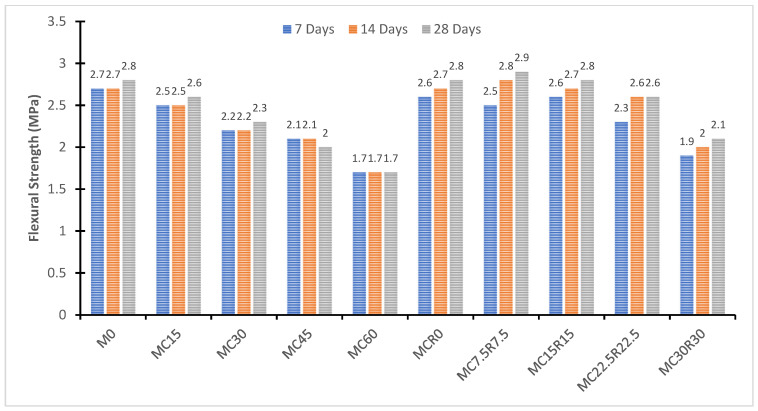
Flexural strength (MPa) of alkali-activated samples at 7, 14, and 28 days.

The test setup for the flexural strength of alkali-activated samples is displayed in Figure 6. The flexural strength of the slag-based GP binder was employed to measure the effectiveness of geo-polymerization. Figure 7 shows the flexural strength variation in the proportion of dehydrated cement paste as a partial replacement for granulated blast furnace slag. As it can be noted from Figure 7, the inclusion of dehydrated cement paste as a partial replacement for cement did not significantly impact the early-age flexural strength of alkali-activated slag-based samples. However, as expected, the later strength of samples improved by a small margin due to the chemical composition of DCP, which helps in the development of extra reaction products, which assist in the later age strength of alkali-activated slag-based samples [62]. The highest strength in later-age curing was 2.8 MPa at 28 days, and the minimal flexural strength was 1.7 MPa in the M60 mix. The low strength can be ascribed to a long-induced period and a slow hydration process. The high strength at a later age can also be attributed to high proportions of silica, magnesium oxide, or calcium oxide [17]. With the addition of both DCP and RM as a partial substitute for GBFS in alkali-activated samples, no significant enhancements in flexural strength were observed except for both 7.5% of DCP and RM in samples, which had 2.9 MPa and could be considered a slight improvement in flexural strength in comparison with the rest of the modified samples. The addition of RM improved the flexural strength compared to the specimens prepared using GBFS and DCP, as shown in Figure 6. In addition, among the specimens proportioned using slag and RM, no significant difference was observed between samples with 7.5% and 15% of RM compared to the control. In contrast, samples made with 22.5% and 30% showed slightly lower flexural strength.

### 3.4. Drying Shrinkage

**Figure 8:**
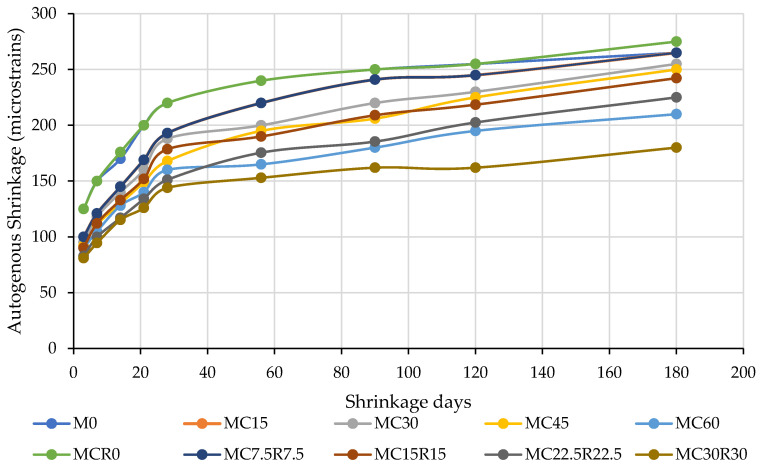
Impact of DCP on dry shrinkage of the slag-based sample.

Figure 8 presents the drying shrinkage values of alkali-activated materials comprising a proportion of dehydrated cement powder. As observed in Figure 8, the drying shrinkage rate was high up to 28 days, and, after the early days, the rate of drying shrinkage did not get as high as it was in the early stages. With the incorporation of 15% dehydrated cement powder, the drying shrinkage at an early stage (3 days) was reduced from 125 to 100 microns. An identical pattern of test outcome was noted for samples assessed in later days, and drying shrinkage values reduced from 100 microns to 90 microns when the amount of DCP was increased from 15% to 60%. Finally, the values of drying shrinkage for alkali-activated materials comprising 60% DCP were decreased from 265 microns to 210 microns, correspondingly. For every dose of alkali-activated materials, it was observed that substituting GBFS with DCP caused the constant reduction in drying shrinkage for all alkali-activated samples. The attained reduction in the values of drying shrinkage with a rising dose of DCP could be attributed to the development of exceptionally interlinked capillary networks inside the paste matrix [63]. The replacement of granulated blast furnace slag with a different dose of dehydrated cement powder led to a variation in the levels of calcium oxide in the paste because of the different levels of calcium oxide in DCP compared to GBFS. Therefore, this variation in the amount of calcium oxide in alkali-activated pastes can reduce the rate of hydration reaction; the samples made with high percentages of DCP displayed reduced drying shrinkage compared to samples with low proportions of DCP [64]. The lower value of drying shrinkage among the first batch mixes of alkali-activated samples was noted to be in the mix “M60”, which can be credited to the quick rates of chemical reaction, which caused a sturdier structure [65] of binder to develop that had high resistance against shrinkage. The presence of sand (fine aggregate) also impacts the shrinkage value of the sample, as the sand does not experience shrinkage. It can also be noted from Figure 8 that as the combined DCP and RM were added as a substitute for GBFS in different percentages, the drying shrinkage was reduced with it. Among all the modified samples at 180 days, the 30% DCP and 30% RM had the lowermost drying shrinkage value of 180 microstrains, which is almost 34.5% lesser shrinkage than the other modified samples.

### 3.5. Resistance against Sulphuric Acid

**Figure 9:**
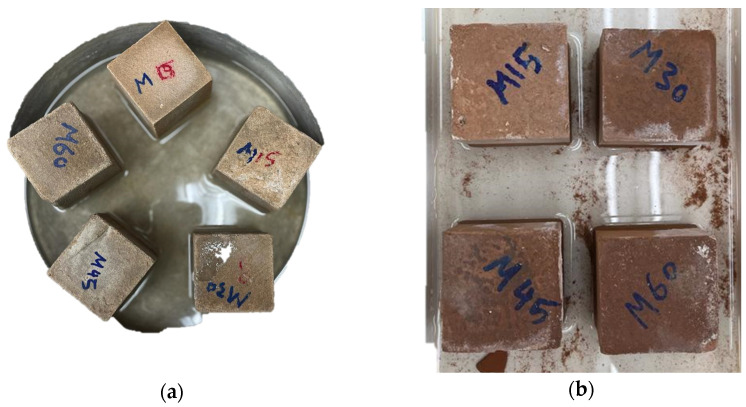
Virtual appearance of alkali-activated samples prepared for acid resistance test (**a**) DCP; (**b**) DCP + RM.

**Figure 10:**
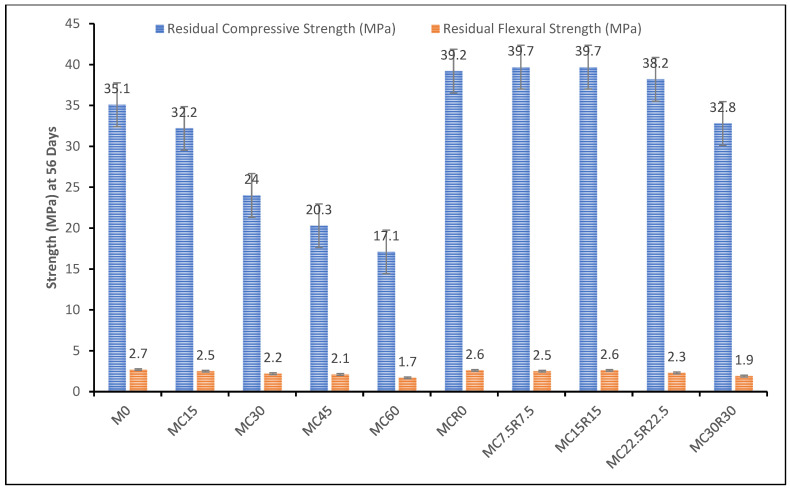
Residual compressive and flexural strength for all mixes after immersion in 10% H_2_SO_4_ solution at 56 days.

Figure 9 shows the alkali-activated samples’ residual compressive and flexural strength when GBFS is replaced with DCP from 0% to 60%. The sulphuric acid resistance test was conducted on samples after 56 days for compressive and flexural residual strength, as shown in Figure 10. From Figure 9, it was observed that as the amount of DCP was increased, the strength gradually reduced constantly as the residual compressive and flexural strength decreased from 35.1 MPa to 17.1 MPa and 2.7 MPa to 1.7 MPa, respectively. Figure 9 shows the samples prepared for the sulphuric acid resistance test. The reduction in strength can be clarified as follows: Initially, calcium-aluminate-silicate-hydrate gel decomposes and decalcifies, causing a considerable loss in strength [17]. Secondly, DCP instigates the expansion of alkali-activated samples, developing extra cracks in the specimen that cause additional wear and tear. As DCP has more calcium oxide, this causes the production of gypsum and a high content of gypsum increases expansion in the sample, increases the size of cracking, enhances spalling, reduces compressive and flexural strength, and also causes a loss in mass [55]. Thus, the specimens having more proportions of DCP led to more deterioration. It can also be observed from Figure 9 that as the combined percentages of DCP and RM were added as a substitute for GBFS in different mixes, the residual compressive and flexural strength showed very noteworthy improvements by almost retaining their compressive and flexural strength when tested after the immersion of samples in a solution of sulphuric acid. This can be ascribed to the high proportion of silica and alumina in red mud, which helps develop the geo-polymerization process and makes the sample’s matrix capable enough to withstand harsh environments such as acid or sulfate attacks [56].

### 3.6. Porosity Test

Parallel to mechanical tests, the porosity of samples was also evaluated at the curing age of 7, 28, and 91 days to confirm the outcome of the mechanical strength of the samples. The sample’s porosity was measured by assessing specimens in different stages of moisture, for example, oven-dried weight (*W_od_*), specimen weight in water (*Ww*), and saturated surface dry weight (*W_ssd_*). The specimen weight in water was evaluated directly after specimens were removed from the curing tank, followed by determining the weight of the saturated surface dry in the open air. Before deciding on the saturated surface dry weight, the surface of the cube samples was cleaned with a cloth to attain the required state of saturated surface dryness. Then, the samples were oven-dried at 103 °C for one day to determine their oven-dried weight. The following formula measured the sample’s porosity:

(1) $$r=\frac{\left(W_{ssd}-W_{od}\right)}{\left(W_{ssd}-W_{od}\right)}\times 100$$

In Equation (1), “*r”* represents the porosity calculated in the percentage.

**Figure 11:**
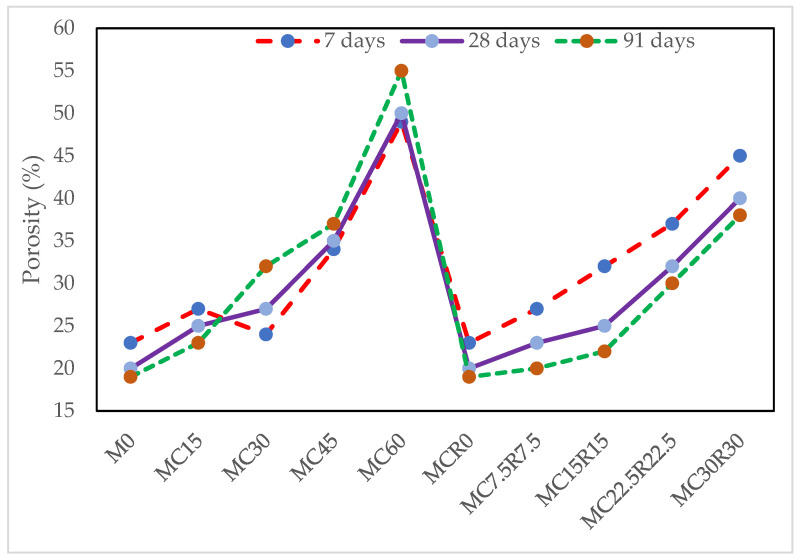
Influence of cementitious waste replacement on the porosity of alkali-activated mortar.

Figure 10 presents the porosity test outcome for alkali-activated samples at 7, 28, and 91 days. From Figure 11, it was observed that the percentage of porosity was high in all samples at early stages, and the highest porosity at an early age (7 days) was noted to be 55%. However, as the curing days increased, the proportion of porosity also reduced with the later ages of the sample, as the porosity reduced by 10.9% for M60 at 91 days compared to the mix M60 at 7 days. The lower porosity shows homogeneity in the binder matrix, which also led to the formation of the strength of the specimen [66]. Regardless of the decrease in the porosity of the sample with age, it was also observed that, with the increment in the amount of DCP as a partial substitute for GBFS in each mix, the porosity would rise with the increase in the percentage of dehydrated cement powder. It can also be noted from Figure 11 that as the combined proportions of DCP and RM were added as a partial replacement for GBFS in different mixes, the porosity continued to rise regardless of the mix type. This could be ascribed to red mud’s ability to absorb huge amounts of water, which leads to more significant porosity. Still, as the curing days increased, the porosity decreased as well. This can be attributed to the very fine particle size of red mud, as studied in past research [67].

### 3.7. Efflorescence

**Figure 12:**
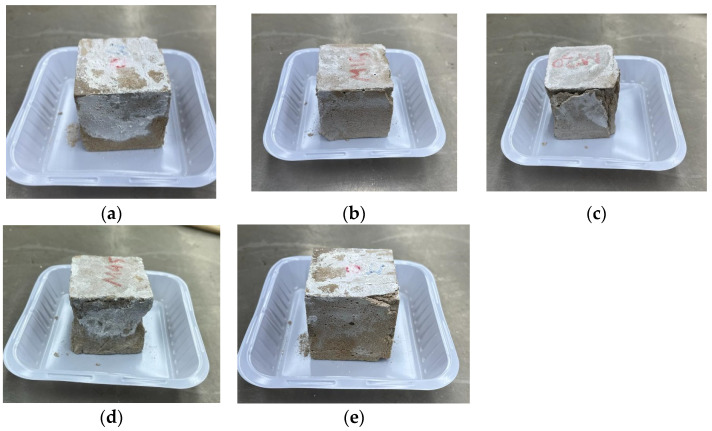
Efflorescence of hardened alkali-activated GBFS paste in contact with water. (**a**) 0%; (**b**) 15% DCP; (**c**) 30% DCP; (**d**) 45% DCP; and (**e**) 60% DCP.

The efflorescence is caused by the relocation of alkalis present in the pore structure to the outer surface, and its reaction with natural carbon dioxide produces sodium carbonate. The rate of efflorescence for every component of the mix was visually observed, as displayed in Figure 12. The observation for the efflorescence test in this study was conducted seven days after the removal of the seal. It was observed that the samples with a higher amount of dehydrated cement powder (DCP) showed more efflorescence. The highest severe efflorescence was noted for the M60 sample. This high efflorescence of the samples can be ascribed to the high porosity of every mix, which causes more water penetration and release of alkali cations [68]. The formation of a more porous microstructure, particularly in the early days, facilitates the leaching of alkalis to the surface. The efflorescence is considered physically harmless.

**Figure 13:**
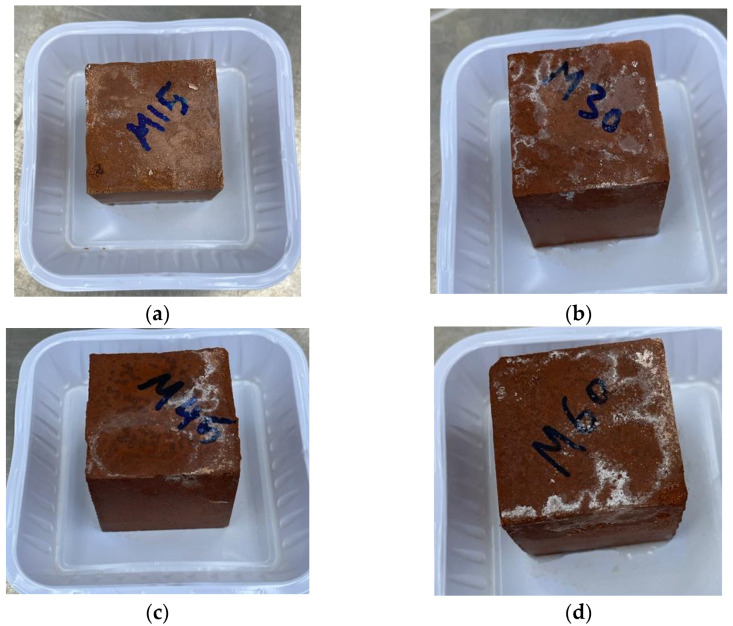
Efflorescence of hardened alkali-activated GBFS sample with both (DCP + RM) in contact with water. (**a**) 7.5% DCP + 7.5% RM; (**b**) 15% DCP + 15% RM; (**c**) 22.5% DCP + 22.5% RM; and (**d**) 30% DCP + 30% RM.

Moreover, the solubility of sodium ions and migration is limited, but the increased movement is because of the heavy dosage of alkaline chemical solution [69]. In Figure 13, DCP and RM were added as a partial substitute for GBFS. As the percentage of red mud increased in the samples, the efflorescence was observed to increase with it. But still, the presence of efflorescence in samples with both DCP + RM was lesser than the samples with only DCP. The efflorescence elements comprised Na^2+^ ions, which come from the red mud’s Na_2_O, which is a primary factor behind the increasing efflorescence. It was reported by Jung et al. [70] and Kwon et al. [71] that increasing the percentage of red mud in the alkali-activated binders instigated an increase in the efflorescence area. Additionally, Kwon et al. [71] observed that the development of efflorescence was highly impacted by the proportion of Na^+^ ions, not Ca^2+,^ existing in the alkali-activated specimens.

## 4. X-ray Dispersive (XRD) Analysis

**Figure 14:**
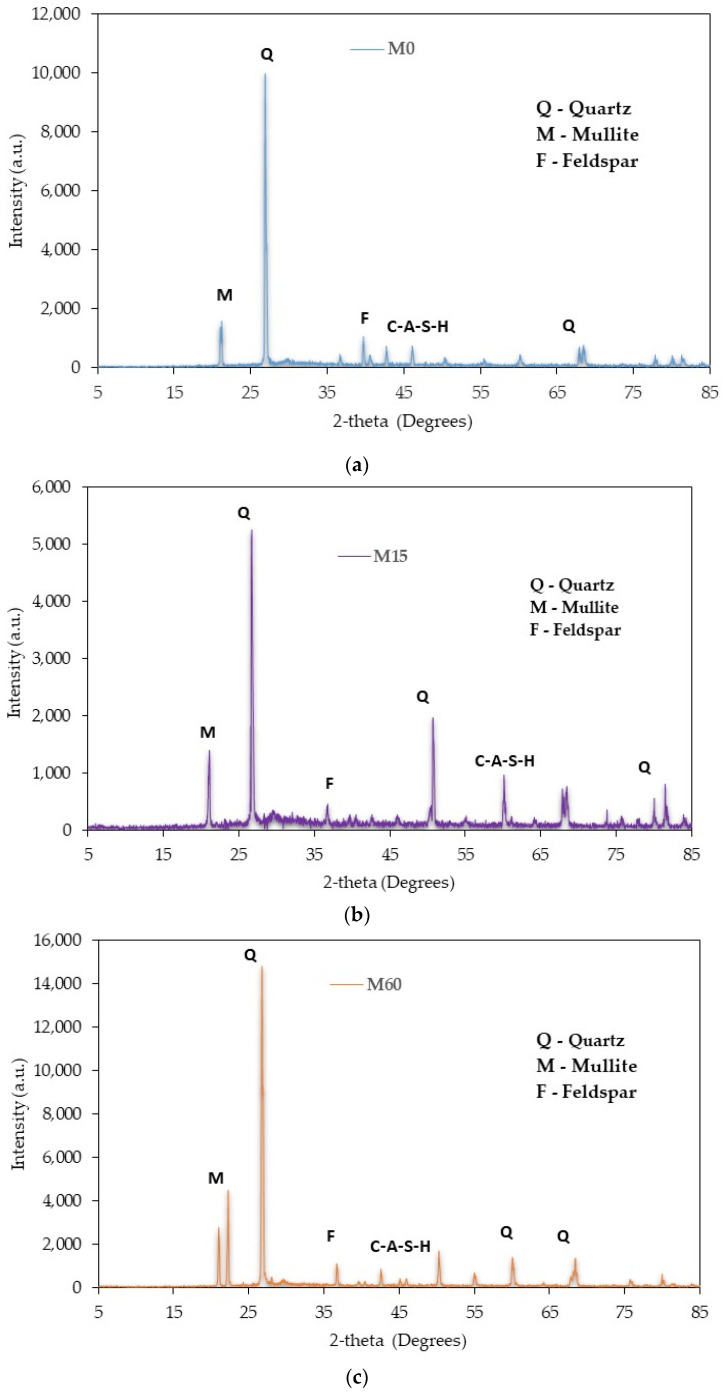
XRD analysis of alkali-activated slag-based samples (**a**) M0; (**b**) M15; and (**c**) M60.

Figure 14a–c shows the XRD analysis of alkali-activated GBFS samples modified with different amounts of dehydrated cement powder (DCP). The presence of glassy phases and the crystallinity of granulated blast furnace slag with DCP favored its solid chemical reaction and produced more reaction products. An amorphous and crystalline quartz (silica) peak was observed at 26 degrees for the M0, M15, and M60 mix, as shown in Figure 14a–c. It should be noted that only amorphous active SiO_2_ contributes to the reaction of polymerization. The peak phase of hydrotalcite could be hard to classify due to its low crystallinity and small content. As the amount of dehydrated cement powder was raised, the intensity of the peak phase for hydrotalcite was also reduced. This can be ascribed to the low infiltration of calcium into the DCP, which caused a decrease in the phase of hydrotalcite. It was also noted that as the amount of DCP was increased, SiO_2_, feldspar, and mullite were spotted, and the peak intensity was raised with the increase in the proportion of DCP.

**Figure 15:**
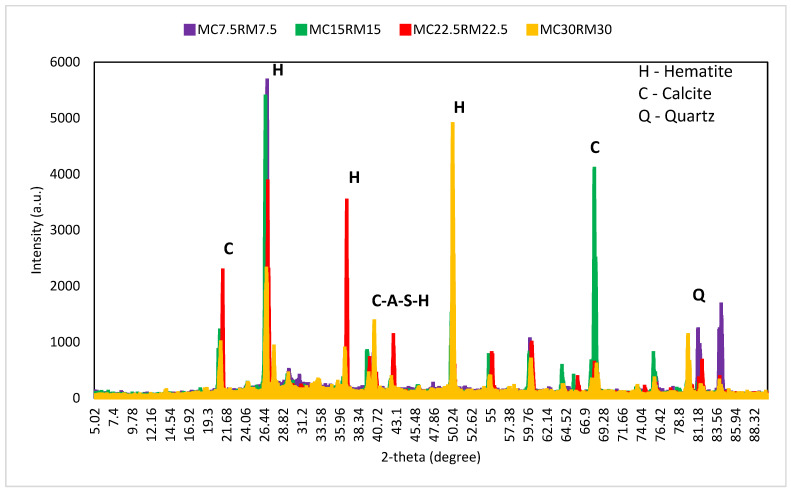
XRD analysis of alkali-activated samples with both DCP and RM.

Furthermore, a phase of calcium-aluminate-silicate-hydrate was also noticed. As calcium-aluminate-silicate-hydrate is an amorphous phase [72], it is quite hard to see the variation in the proportion of calcium-aluminate-silicate-hydrate through various specimens. Figure 15 presents the combined X-ray diffraction analysis of samples with dehydrated cement powder and red mud as a partial substitute for GBFS in the alkali-activated samples. Increasing the proportion of red mud led to the broad, amorphous peak, which signifies the development of amorphous gel products. This indicated that the crystalline stages in red mud did not partake in the process of geo-polymerization but instead behaved as a filler in the geopolymer’s matrix [73]. It was also observed that when the amount of red mud was increased, the geopolymer structure’s development process slowed, which implies that red mud was partially involved in the hydration of the binder’s process. It was also observed that, regardless of the proportion of red mud, every sample showed a similar phase composition but was different in their peaks, as presented in 15. RM depicts a sharp peak from calcite and hematite, suggesting that the amorphous phase is not a high proportion. Additionally, crystal phases are in the lead in RM, which is a non-calcined material. The peak phases are more pronounced in Figure 15, which signifies a greater extent of geo-polymerization and more geopolymer binding material present in pure form in the samples than the samples with only GBFS or DCP. The sample with 22.5% DCP and 22.5% RM showed a bit more crystalline phases than the other mixes, which could be interpreted as a specific mix with a high degree of in-active filling materials and a low proportion of geopolymer binding material.

## 5. Scanning Electron Microscopy (SEM) Analysis

**Figure 16:**
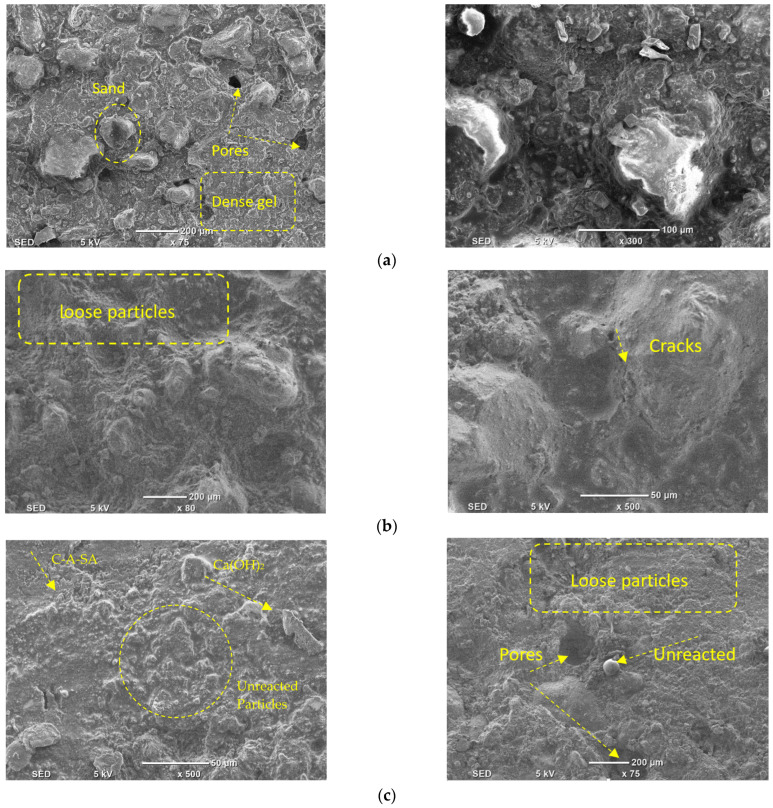
SEM analysis of alkali-activated slag-based samples with different levels of DCP. (**a**) 0%; (**b**) 15%; and (**c**) 60%.

Figure 16 displays the SEM micrographs of alkali-activated slag-based samples with different proportions of DCP, ranging from 0% and 15% to 60%. The development of a more robust bond amid the partly reacted DCP was observed. The sample with no DCP did not show any cracks in SEM images. Though the amount of DCP was raised from 0% to 60%, the network of polymer bonds gets weak, which leads to structural wear and tear through the development of more pores. With raising the amount of DCP, the concentrations of particles that were non-reactive were observed to be increased, and the development of calcium-aluminate-silicate-hydrate and calcium-silicate-hydrate gel products [74] was impacted by the existence of the huge content of silicates and reduced proportions of calcium, which leads to the formation of less packed gels of hydrotalcite and mullite in comparison to the gel of calcium-aluminate-silicate-hydrate [75].

**Figure 17:**
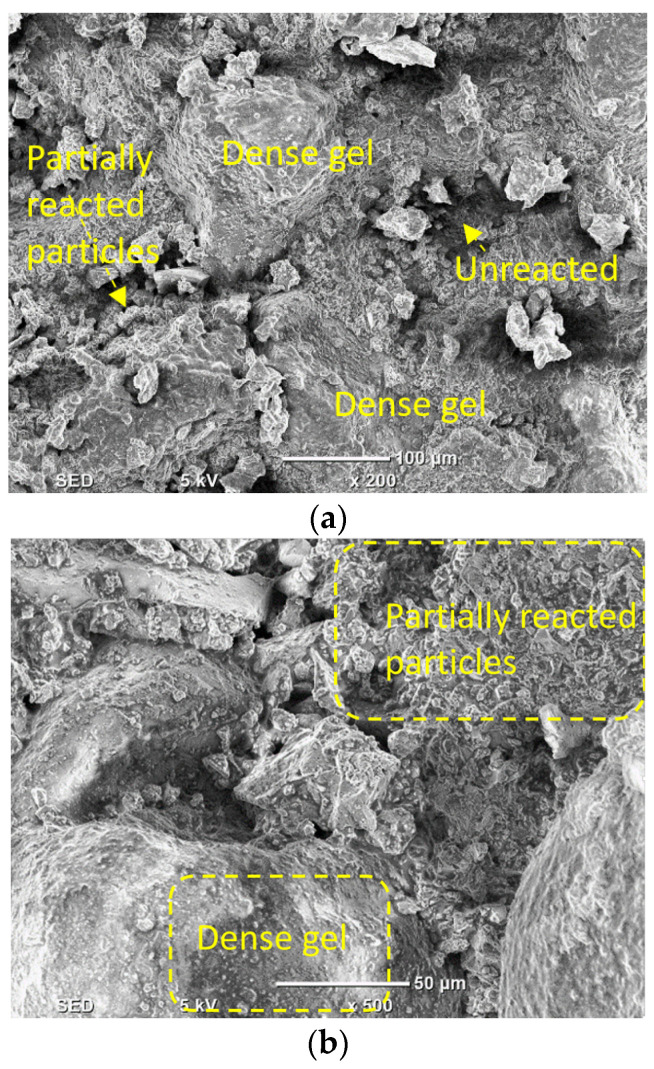
SEM micrograph of alkali-activated samples with both DCP and RM (**a**) MC7.5RM7.5; (**b**) MC30RM30.

Furthermore, a considerable proportion of SiO_2_ is left unreactive. The less-dense microstructure of alkali-activated samples with a large amount of DCP (60%) was observed to have the lowest mechanical strength at all curing ages. Figure 16 presents the SEM micrographs of samples (MC7.5RM7.5 and MC30RM30) with both DCP and RM as partial substitutes of GBFS in the alkali-activated samples. In Figure 17, a porous microstructure can be noticed. Different attributes can be observed. Firstly, Figure 17a,b shows micro-size voids and cracks. The profusion of micro-cracks could be instigated by two motives: (I) some of the cracking could be introduced by drying shrinkage of the samples during curing when the water evaporated; (II) the specimens tested under a scanning electron microscopic test were loaded to failure under a compressive strength test; the cracking could have been introduced by the applied load. The huge volume of micro-pores and micro-cracks has a detrimental impact on the mechanical characteristics and durability of the alkali-activated sample.

## 6. Sustainability Perspective of DCP

As dehydrated cement powder is a waste material, utilizing it in a sample will lead to a reduced outflow of CO_2_ into the atmosphere. Past studies have shown that samples with only cement as a primary binder would lead to 380 kg of CO_2_ per unit volume, and when DCP was incorporated into the samples, the CO_2_ dropped to 297 kg per unit volume. Usually, all the phenomena stated above could be ascribed to the following reasons: (a) Dehydrated cement powder is formed from discarded cementitious materials, and it requires low energy and resources during the process of its production in comparison to cement. (b) Dehydrated cement powder has binding properties and a significant content of finer particles, which may assist in a chemical reaction. During the lab tests, it was observed that dehydrated cement powder could be utilized as an adequate substitute for GBFS, but it should be used in a specific amount. Including dehydrated cement powder can decrease the cost of the overall sample-making process and its influence on the atmosphere. From an environmental and economic point of view, it can be decided that dehydrated cement powder is a suitable waste cementitious material that can be utilized effectively to improve the properties of the sample, and it will also reduce the land dumping of DCP, which will ultimately help in bringing sustainability to the construction industry [76].

## 7. Conclusions

This research studied the effects of adding dehydrated cement powder (DCP) as a partial replacement for GBFS in developing alkali-activated slag-based mixtures. Different strength and durability tests were carried out, and microstructural analysis was performed. The following conclusions were obtained from this study;

1. As the amount of DCP increased, the mixes’ flowability decreased. With both DCP and RM, the flowability rose to a specific level and then decreased. This is due to the different chemical and physical reactions of raw ingredients.
2. The mechanical strength of alkali-activated samples was deceased with a rise in the proportion of DCP utilized as a substitute for GBFS from 0% to 60%. The compressive strength was enhanced by 42.2% at 28 days when RM was added to DCP.
3. At each level of DCP, the substitution of GBFS with DCP induced a loss in strength due to the detrimentally impacted formulation of calcium-aluminate-silicate-hydrate gel, which ultimately caused a drop in mechanical strength.
4. The residual compressive and flexural strength displayed an improvement in the alkali-activated sample’s resistance against acid attack with an increase in DCP. This was prevented significantly when the sample had DCP and RM, with samples almost retaining their original strength values.
5. The outcome of the XRD analysis showed that the formation of ettringites and gypsum was constrained by the rise in the amount of DCP as a partial substitute for GBFS in the mixture. Additionally, the development of crystalline phases was observed due to the addition of RM to DCP, which helped in the process of geo-polymerization.
6. The study showed that DCP could be used up to a certain level to improve the durability of alkali-activated samples, as observed in the drying shrinkage test. The shrinkage was observed to decrease continuously with adding RM along with DCP. As the testing age increased, shrinkage decreased up to 180 days.
7. It is recommended to utilize DCP at a low dosage because, as observed in the study, both the level of porosity and efflorescence increased with the rising level of DCP. Due to the addition of RM, the degree of efflorescence development was better controlled.

Through the methodical preparation of novel alkali-activated mixtures and their systematic classification, it was confirmed that dehydrated cement powder (DCP) and red mud (RM) could provide flexibility to attain good mechanical and durability characteristics and satisfy the sustainability requirements of the construction sector. It was demonstrated that the exploitation of this novel alkali-activated mixture could advance both the economic and sustainability agendas of the construction sector.

## Figures and Tables

**Table 1:** Chemical composition of GBFS and DCP.

Oxide Composition	RM (%)	GBFS (%)	DCP (%)
Na_2_O	3.87	0.23	0.14
CaO	11.5	46.1	52.2
SiO_2_	19.6	30.6	19.9
MgO	0.28	5.08	2.91
Al_2_O_2_	14.5	13.67	5.0
Fe_2_O_3_	29.5	0.33	4.125
SO_3_	2.27	1.35	2.93
K_2_O	-	0.36	0.78
TiO_2_	12.3	0.4	0.36
MnO	-	1.69	-
Total	93.82	99.71	88.34
Ignition Loss	6.18	0.29	11.66

RM—Red Mud; GBFS—Granulated Blast-Furnace Slag; DCP—Dehydrated Cement Powder.

**Table 2:** Mix the proportion of samples (g/cm^3^).

Mix ID	GBFS (g)	CW Substitution (g)	RM Substitution (g)	Water	NaOH	Sodium Silicate	Sand (g)
M0	1000	0	0	395	36.01	253.0	2256
MC15	850	150	-	395	36.01	253.0	2280
MC30	700	300	-	395	36.01	253.0	2303
MC45	550	450	-	395	36.01	253.0	2326
MC60	400	600	-	395	36.01	253.0	2326
MCR0	1000	0	0	395	36.01	253.0	2256
MC7.5R7.5	850	75	75	395	36.01	253.0	2280
MC15R15	700	150	150	395	36.01	253.0	2303
MC22.5R22.5	550	225	225	395	36.01	253.0	2326
MC30R30	400	300	300	395	36.01	253.0	2326

## Data Availability

Not applicable.
